# Comparing outcomes from tailored meta-analysis with outcomes from a setting specific test accuracy study using routine data of faecal calprotectin testing for inflammatory bowel disease

**DOI:** 10.1186/s12874-022-01668-9

**Published:** 2022-07-12

**Authors:** Karoline Freeman, Brian H. Willis, Ronan Ryan, Sian Taylor-Phillips, Aileen Clarke

**Affiliations:** 1 Division of Health Sciences, University of Warwick, Coventry, CV4 7AL UK; 2 Institute of Applied Health Research, University of Birmingham, Birmingham, B15 2TT UK

**Keywords:** Meta-analysis, Routine data, Diagnostic test accuracy, Decision making

## Abstract

**Background:**

Meta-analyses of test accuracy studies may provide estimates that are highly improbable in clinical practice. Tailored meta-analysis produces plausible estimates for the accuracy of a test within a specific setting by tailoring the selection of included studies compatible with a specific setting using information from the target setting. The aim of this study was to validate the tailored meta-analysis approach by comparing outcomes from tailored meta-analysis with outcomes from a setting specific test accuracy study.

**Methods:**

A retrospective cohort study of primary care electronic health records provided setting-specific data on the test positive rate and disease prevalence. This was used to tailor the study selection from a review of faecal calprotectin testing for inflammatory bowel disease for meta-analysis using the binomial method and the Mahalanobis distance method. Tailored estimates were compared to estimates from a study of test accuracy in primary care using the same routine dataset.

**Results:**

Tailoring resulted in the inclusion of 3/14 (binomial method) and 9/14 (Mahalanobis distance method) studies in meta-analysis. Sensitivity and specificity from tailored meta-analysis using the binomial method were 0.87 (95% CI 0.77 to 0.94) and 0.65 (95% CI 0.60 to 0.69) and 0.98 (95% CI 0.83 to 0.999) and 0.68 (95% CI 0.65 to 0.71), respectively using the Mahalanobis distance method. The corresponding estimates for the conventional meta-analysis were 0.94 (95% CI 0.90 to 0.97) and 0.67 (95% CI 0.57 to 0.76) and for the FC test accuracy study of primary care data 0.93 (95%CI 0.89 to 0.96) and 0.61 (95% CI 0.6 to 0.63) to detect IBD at a threshold of 50 μg/g. Although the binomial method produced a plausible estimate, the tailored estimates of sensitivity and specificity were not closer to the primary study estimates than the estimates from conventional meta-analysis including all 14 studies.

**Conclusions:**

Tailored meta-analysis does not always produce estimates of sensitivity and specificity that lie closer to the estimates derived from a primary study in the setting in question. Potentially, tailored meta-analysis may be improved using a constrained model approach and this requires further investigation.

## Background

Meta-analyses of sensitivity and specificity combine results from several independent studies. This is an advantage over single test accuracy studies particularly in the evaluation of tests for rare diseases where studies of test accuracy are often small. As a result, outcomes of test accuracy from meta-analysis are considered to be more precise and may provide insights into the consistency of test results [1]. However, the disadvantage of meta-analyses is that they provide an average of the sensitivity and specificity. These may not be sufficiently applicable to a specific population or setting of interest because the estimates were derived from heterogeneous studies in terms of patient population and settings. This may impede local decision-making on test use or patient management.

The tailored meta-analysis addresses this problem of conventional meta-analyses by combining setting-specific information with evidence from systematic reviews to produce more relevant outcomes for the setting of interest [2, 3]. The aim of the tailored approach is to define an applicable region in the receiver operating characteristic (ROC) space which is a plausible range of values for the sensitivity and specificity of the test in the setting of interest informed by its test positive rate and prevalence. The applicable region can then be used to determine which of the eligible studies are truly relevant to the setting of interest and should be considered for meta-analysis. This has been demonstrated for the performance of tests in cancer screening programmes in the UK context and for diagnostic tests in individual general practices [2, 3]. In most of the published examples tailored meta-analysis produced different results which were believed to be more applicable to the specific setting than results from conventional meta-analyses. Differences were sufficiently large to suggest they may lead to different decisions in patient management.

However, to date the results from tailored meta-analysis have not been compared to a primary study in the setting in question. Such comparison is needed in order to validate the tailored meta-analysis approach and determine how close it may come to the “true” accuracy. The aim of this study was to test the hypothesis that the results of the tailored meta-analysis are closer to the study outcomes of a test accuracy study in the setting of interest than the results of a conventional meta-analysis.

We used faecal calprotectin (FC) testing for the diagnosis of inflammatory bowel disease (IBD) in patients with chronic abdominal symptoms as an example in this validation study. Faecal calprotectin is an inflammatory maker that can be measured in stool samples. Levels above 50 μg/g are typically classified as positive indicating a referral to gastroenterology for confirmatory testing. The aim of the study was to estimate test accuracy of FC testing in primary care using tailored meta-analysis and compare this to primary care estimates.

## Methods

### Tailored meta-analysis

The current model of tailored meta-analysis relies on four steps. Firstly, data on the test positive rate and disease prevalence need to be collected from the setting in question. Secondly, this is used to derive an applicable region for the test in the setting. Thirdly, test accuracy studies of the test need to be identified using systematic review methods and the sensitivities and false positive rates reported in the studies compared with the applicable region to aid the selection of studies for meta-analysis [2, 3]. Finally, the selected studies are meta-analysed. These steps are described next in more detail.

#### Data collection for the test positive rate and disease prevalence from the primary care setting

We used The Health Improvement Network (THIN), a database of routine electronic health records from UK primary care, to determine the FC test positive rate and IBD prevalence for primary care. In a retrospective cohort study of adult patients (≥18 years) all patients with a first FC test recorded between 2006 and 2016 were identified. IBD was defined as a clinical code for IBD and its sub-conditions or a code for an IBD specific prescription. The test positive rate was defined as the proportion of FC tests with a numeric value of > 50 μg/g. Prevalence was defined as the proportion of patients with an IBD record in the FC tested population. 99.98% confidence intervals for test positive rate and prevalence were calculated using the Hotelling method which takes into consideration the correlation between the prevalence and test positive rate [4].

#### Defining the applicable region

The applicable region in the ROC space resembles the area of sensitivity and false positive rate (1-specificity) pairs for FC testing that are feasible for the primary care setting. We plotted the applicable region using the mathematical relationship between the test positive rate, the prevalence, the sensitivity and the false positive rate described by Willis et al. [2, 3].

#### Primary studies from a systematic review of test accuracy and selection of studies for tailored meta-analysis

We included primary test accuracy studies identified in our independent systematic review and meta-analysis of faecal calprotectin for the detection of inflammatory bowel disease which included studies from secondary and primary care [5].

Studies with test accuracy estimates falling within the derived applicable region were considered applicable for the primary care setting. Studies with estimates falling outside the applicable region were assessed for the feasibility of their true population parameters to lie within the applicable region following methods already described [2]. In brief, we chose the point on the boundary that is most likely to represent the true parameter for an individual study conditional on it lying in the applicable region. To estimate the boundary parameter, two approaches have been proposed. The first uses a maximum likelihood estimate for the parameter after assuming the sensitivity and false positive rate follow independent binomial distributions. The second uses an estimate which minimises the Mahalanobis distance between the boundary and the study as previously described [2]. Study selection is then based on comparing the observed sensitivity and false positive rate with the boundary parameter using an appropriate statistical test. Where the probability was smaller than 0.025 the study was rejected.

#### Statistical analysis

For the meta-analysis we considered studies of test accuracy of FC testing for IBD at a 50 μg/g threshold to derive summary estimates of sensitivity and specificity. We undertook a bivariate random-effects meta-analysis (BRM) [6] including only studies that were plausible to fall within the applicable region, i.e. that were compatible with the test positive rate and prevalence found in primary care. All analyses were undertaken in R version 3.6.1 [7].

### Test accuracy study in primary care

Estimates of sensitivity and specificity from the meta-analysis were compared to estimates from an independent primary care test accuracy study using the same primary care THIN dataset. Details of the study are published elsewhere [8]. In brief, in our analysis we considered 5970 patients with at least 6 months of follow-up data (for an analysis where an IBD diagnosis was considered when it was recorded within 6 months of the FC test) of the 7084 patients we identified who had had an FC test and no prior IBD diagnosis since registration with the general practice. The target condition was IBD recorded as a clinical code or a code for an IBD specific prescription within 6 months of FC testing. Disease negatives were defined as not having an IBD record. An FC test was classified as positive if the numeric result was > 50 μg/g.

### Comparison of meta-analytical results with estimates from a primary test accuracy study

In this comparison we considered plausibility as well as closeness. We were interested in whether estimates from tailored and conventional meta-analysis were in a plausible region defined by the test positive rate and prevalence of the target condition in the setting of interest. When measured to 99% confidence this constrains the region in ROC space of plausible estimates in ROC space. Since the probability of the applicable region containing the ‘true’ sensitivity and false positive rate is greater than 99%, the probability that the rest of ROC space outside of the applicable region contains the ‘true’ sensitivity and false positive rate is less than 1%. Thus, estimates outside of the applicable region are highly unlikely to be representative of the test in the setting.

Closeness was assessed using a geometrical measure (the Euclidean distance) which quantifies the physical distance between two points due to the bivariate nature of the analysis.

Given this, an estimate may be geometrically closer than another estimate but if it lies in the region outside the applicable region there is less than 1% probability that it or any other estimate in that region could represent the true sensitivity and specificity for the setting.

## Results

### Test positive rate and IBD prevalence to determine the applicable region in ROC space

**Fig. 1:**
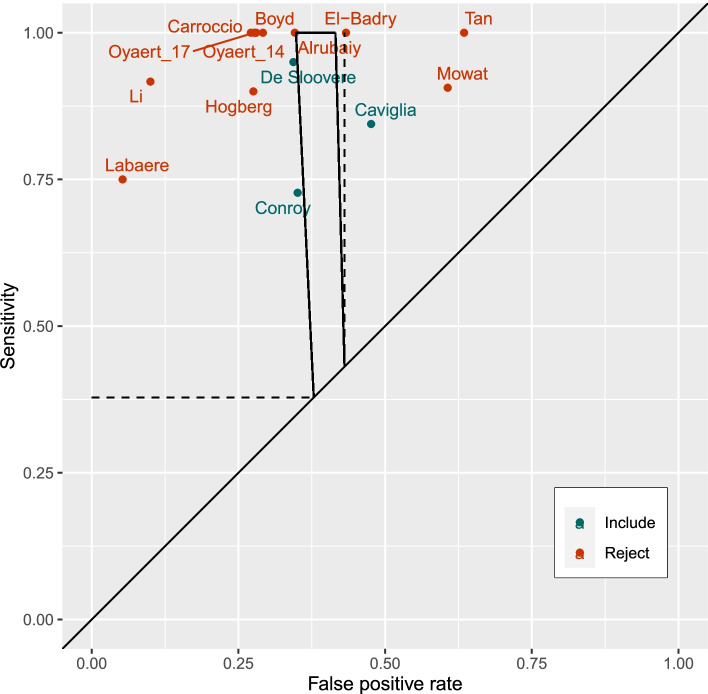
ROC plot of studies reporting sensitivity and specificity of FC testing for IBD at 50 μg/g (binomial method). The applicable region for primary care is defined by the test positive rate (dashed line) and by test positive rate plus prevalence (trapezium) from THIN data defines the area of sensitivity and specificity that is compatible with UK primary care practices. Included studies using the binomial distribution method (Caviglia 2014 [9], Conroy 2018 [10] and DeSloovere 2017 [11]) were compatible with their true parameters lying in the applicable region unlike the rejected studies (Alrubaiy 2012 [12], Boyd 2016 [13], Carroccio 2003 [14], El Badry 2010 [15], Hogberg 2017 [16], Labaere 2014 [17], Li 2006 [18], Mowat 2016 [19], Oyaert 2017 [20], Oyaert 2014 [21] and Tan 2016 [22])

In a dataset of 7084 first time FC tests the test positive rate was 40.4% (99.98% CI 37.8 to 43.1%) and the IBD prevalence was 3.5% (99.98% CI 2.7 to 4.6%). The applicable region in ROC space based on these estimates is shown in Fig. 1.

### Selection of studies for tailored meta-analysis

The published review [5] included 14 studies from primary and secondary care evaluating faecal calprotectin for the differentiation of IBD and non-IBD at the FC threshold of 50 μg/g [9–22]. An analysis of test accuracy by setting was not feasible because of heterogeneity within the small number of primary care studies. Furthermore, categorisation of studies into primary and secondary care was mainly arbitrary because the study populations were often mixed, highly selected or referred.

**Fig. 2:**
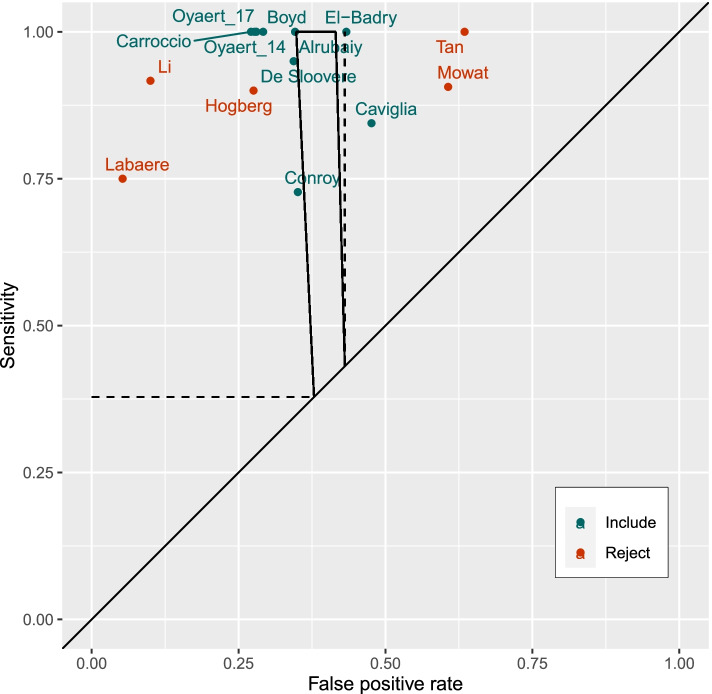
ROC plot of studies reporting sensitivity and specificity of FC testing for IBD at 50 μg/g (Mahalanobis distance method). The applicable region for primary care is defined by the test positive rate (dashed line) and by test positive rate plus prevalence (trapezium) from THIN data defines the area of sensitivity and specificity that is compatible with UK primary care practices. Included studies using the Mahalanobis distance method (Carroccio 2003 [14], Oyaert 2014 [21], Oyaert 2017 [17], Boyd 2016 [13], Alrubaiy 2012 [12], DeSloovere 2017 [11], Conroy 2018 [10], El Badry 2010 [15], and Caviglia 2014 [9]) had closer ‘statistical distance’ to the applicable region than rejected studies (Labaere 2014 [17], Li 2006 [18], Hogberg 2017 [16], Mowat 2016 [19] and Tan 2016 [22])

Figures 1 and 2 show the 14 studies in the ROC space and in relation to the applicable region identifying the area of greatest plausibility for UK primary care. None of the study estimates lay in the applicable region which was narrow due to the precision when using large datasets. Using the binomial method for study selection, 11 of the studies had a low probability of producing the study estimate given that the study parameter lay on the boundary (Fig. 1) [12–22]. These studies were excluded from meta-analysis because they were outside the range of performances feasible for UK primary care practices as defined by the THIN data.

Figure 2 shows the results of study selection using the Mahalanobis distance method. Using this method, only five studies are excluded as being incompatible with the applicable region [16–19, 22]. However, 6 of the included studies reported sensitivities of 100%, that is on the boundary of ROC space.

### Tailored meta-analysis and comparison of test accuracy with outcomes from primary care

The results of the tailored meta-analyses in comparison to the results from conventional meta-analysis and the primary care study are shown in Table 1. Sensitivity and specificity from tailored meta-analysis using the binomial method were 0.87 (95% CI 0.77 to 0.94) and 0.65 (95% CI 0.60 to 0.69); however, there were only 3 included studies. In contrast, tailored meta-analysis using the Mahalanobis distance method included 9 studies and the sensitivity and specificity were 0.98 (95% CI 0.83 to 0.999) and 0.68 (95% CI 0.65 to 0.71), respectively. The corresponding estimates for the conventional meta-analysis were 0.94 (95% CI 0.90 to 0.97) and 0.67 (95% CI 0.57 to 0.76).

**Table 1:** Comparison of sensitivity and specificity from tailored meta-analysis, conventional meta-analysis and results using THIN data

	Sensitivity (95% CI)	Specificity (95% CI)	Euclidean distance (compared to THIN estimate)	In applicable region yes/no
Primary Care (THIN)	0.93 (0.89 to 0.96)	0.61 (0.60 to 0.63)	0	Yes
Conventional MA (14 studies)	0.94 (0.90 to 0.97)	0.67 (0.57 to 0.76)	0.059	No
Tailored MA (binomial) (3 studies)	0.87 (0.77 to 0.94)	0.65 (0.60 to 0.69)	0.062	Yes
Tailored MA (Mahalanobis) (9 studies)	0.98 (0.83 to 0.999)	0.68 (0.64 to 0.72)	0.088	No

Comparison of sensitivity and specificity at the common threshold of 50 μg/g

*MA* meta-analysis, *THIN* the health improvement network

The FC test accuracy study of primary care data reported a sensitivity of 0.93 (95%CI 0.89 to 0.96) to detect IBD at a threshold of 50 μg/g. Specificity was 0.61 (95% CI 0.6 to 0.63).

**Fig. 3:**
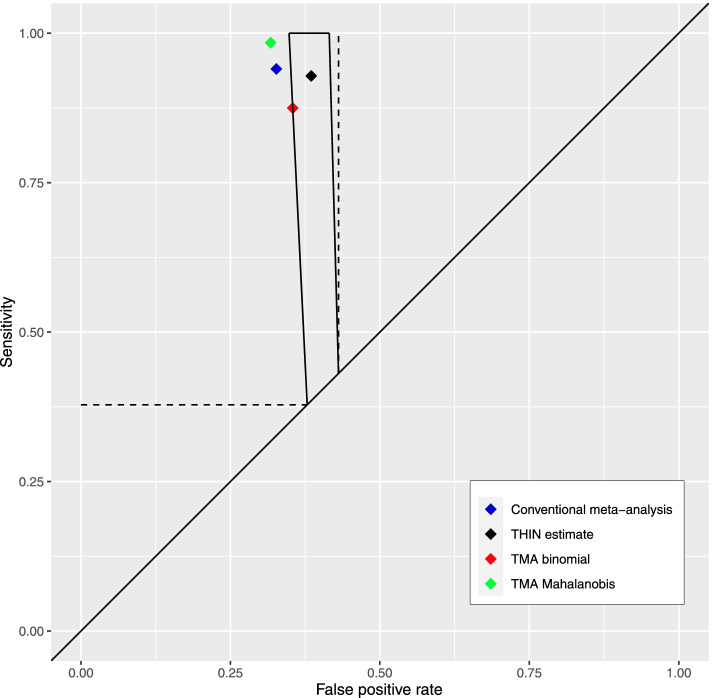
Sensitivity and false positive rate pairs in ROC space from conventional meta-analysis, tailored meta-analysis and THIN data. Tailored meta-analysis was undertaken using the binomial and Mahalanobis distance methods. The applicable region (trapezium) was informed by routine data from primary care. TMA tailored meta-analysis, THIN the health improvement network

While confidence intervals overlapped (Table 1), from the three meta-analysis point estimates only the tailored meta-analysis point estimate using the binomial method was in or on the boundary of the applicable region (Fig. 3). However, there is substantial uncertainty with this estimate given it was synthesised from only 3 studies. In terms of Euclidean distance (Table 1), the conventional meta-analysis estimate is the closest to the THIN estimate but is outside the applicable region and therefore improbable. The tailored estimate using the Binomial method compared with the conventional estimate is marginally more distant in terms of Euclidean distance but as it is on the boundary of the applicable region remains like all others in the applicable region, a plausible estimate, whereas the tailored estimate using the Mahalanobis distance is both the most distant and is outside of the applicable region – therefore is highly improbable.

Th﻿is is most likely due to the majority of studies included being small (wide confidence intervals) and lying on the left-hand side of the narrow applicable region.

## Discussion

### Summary of study findings

We scrutinised two methods of tailored meta-analysis. Of the 14 test accuracy studies identified for conventional meta-analysis three were deemed to be applicable to the primary care setting based on tailoring using the binomial method and nine when using the Mahalanobis distance method. None of the included studies lay in the applicable region. Two [10, 13] of the three primary care studies [10, 13, 16] were included in the tailored meta-analysis using the Mahalanobis distance method but only one [10] was included with the binomial method. This demonstrates that superficial equivalence of the setting does not guarantee that the performance statistics of a test are actually applicable to the setting of primary care defined by data from routine primary care electronic health records using this model. The tailored meta-analysis of nine studies resulted in estimates of sensitivity and specificity of 0.98 (95% CI 0.83 to 0.999) and 0.68 (0.65 to 0.71). The estimates were further away from the THIN estimate of 0.93 (95% CI 0.89 to 0.96) and 0.61 (95% CI 0.6 to 0.63) than the estimates from conventional meta-analysis including all 14 studies. The tailored meta-analysis of three studies produced estimates of sensitivity and specificity of 0.87 (0.77 to 0.94) and 0.65 (0.60 to 0.69) which lay on the boundary of the applicable region. However, most of the evidence was rejected using the binomial method. The study presents an example where the tailored results were not closer to the primary test accuracy study estimates than the result from conventional meta-analysis. However, estimates were close and confidence intervals overlapped.

### Study limitations

The tailored result is based on more information than the conventional result because it combines information from published studies with information from the setting in question and should, therefore, be closer to the estimate from the primary study. Our findings disagree with this expectation as in this example half (7/14) of the studies reported 100% sensitivity. These studies’ estimates are on the boundary of the ROC space which exposed limitations of the two methods. The Mahalanobis distance method assumes the sensitivity and false positive rate have normal distributions and so we use the normal approximation for the variance of a proportion. This is reasonable when the sensitivity and false positive rate are in the 10-90% range but is not an accurate approximation when on the boundary of the ROC space where the sensitivity and false positive rate are either 1 or 0. Therefore, this method is likely to be less accurate for extreme studies on the boundary of ROC space. This is compounded by the calculation of the Mahalanobis distance (D) where we divide by the variance which when using the normal approximation to a sample proportion is estimated to be zero on the boundary. Thus when the sensitivity equals 0 or 1 or the false positive equals 0 or 1 this makes D infinite. To avoid this we only consider points > 0.01 or < 0.99 so the Mahalanobis distance remains finite. Therefore, the Mahalanobis distance method does not deal with points on the actual boundary of ROC space. Statistically, the binomial method is preferred over the Mahalanobis distance method. However, in this example the binomial method resulted in the exclusion of 11/14 studies including all seven studies with 100% sensitivity. This was because the approach uses the binomial distribution to estimate cumulative probabilities where probabilities are bound at one, therefore the cumulative probability is zero for studies where the observed sensitivity or specificity is 1, and the studies are subsequently excluded. This reveals limitations of the current model of tailored meta-analysis which excludes studies deemed implausible and uses the standard BRM model for estimating the sensitivity and specificity. Furthermore, if most of the included studies are on one side of the applicable region this increases the chance of the summary estimate to lie outside the applicable region as we demonstrated here with the Mahalanobis distance method. A potential solution is to include all studies but incorporate the constraints in the BRM to produce a constrained model. The constrained model was shown to be more likely to yield a plausible estimate for the sensitivity and specificity in the practice setting than an unconstrained model [23]. However, this requires further investigation.

The success of the tailored meta-analysis method relies on the fact that the applicable region is correct. This requires accurate estimates of the test positive rate and IBD prevalence. However, the test positive rate using the primary care data is slightly greater compared to those reported in primary care FC test accuracy studies (data not shown). Furthermore, there was a great proportion of FC tests with missing results. It may be possible that positive test results are recorded with more diligence than test negative results in primary care practice which in turn would result in higher test positive rates. This creates some uncertainty about the estimate of the test positive rate used in the tailored meta-analysis.

The IBD prevalence relies on accurate and complete coding of IBD in primary care records. However, potentially missing codes could not be identified or quantified which casts some doubt on the reliability of the prevalence of IBD used to define the applicable region. However, IBD prevalence in FC tested patients (4.2%) was within the range of prevalence estimates reported in seven primary care studies (range 2.7-6.3%) [10, 13, 16, 24–27].

Uncertainty in estimates of test positive rate and prevalence may have led to incorrect boundaries being drawn for the applicable region. However, this was mitigated, as suggested by Willis and Hyde 2014 [3], by using 99.98% CI intervals with high coverage probability to maximise the probability of studies being included.

In this comparison, the tailored results were compared to the ‘true’ estimates for primary care from an independent study of routine primary care data [8]. That study may or may not be biased. However, considering all strengths and limitations discussed previously [8], it probably represents the best estimate we are likely to achieve on the test performance of faecal calprotectin as it is used in UK primary care without conducting a de novo cross sectional study under tightly controlled study conditions.

We considered a calprotectin threshold of 50 μg/g. However, other thresholds have been suggested [28]. The size and position of the applicable region for tailored meta-analysis is determined in part by the test positive rate – the point estimates were 0.4 when the threshold was 50 μg/g and 0.12 when the threshold was 250 μg/g. Since the test positive rate depends on the threshold, the position of the applicable region shifts as the threshold changes. A change in the position of the applicable region would likely affect the composition of the included studies in the tailored meta-analysis and hence the tailored estimates for the sensitivity and specificity. Specifically, as the threshold increases, the test positive rate decreases, and the applicable region shifts down the sensitivity/false positive rate line towards the sensitivity axis. This is more likely to yield estimates with a high specificity and low sensitivity although this would depend on the composition of the studies.

### Findings in context of published evidence

The estimates from tailored meta-analysis and conventional meta-analysis were similar considering their confidence intervals. This may be because the applicable region excluded studies on both sides of its boundaries. Since both estimates represent an average of the included studies excluding studies from both sides did not have a significant impact on the averages. This is in contrast to tailored meta-analyses in the literature where studies outside the applicable region were either all outside the left boundary or all outside the right boundary of the applicable region [2, 3]. Furthermore, examples in the literature all had some studies falling into the applicable region. This was true for narrow applicable regions informed by routine data from UK screening programmes as well as wider applicable regions informed by limited UK data from a single primary care practice. In these previous examples, estimates from tailored meta-analysis fell within the applicable region and presented better estimates for the setting of interest. However, none of the published studies compared the tailored and conventional result with an estimate from the setting itself as data for the disease status following testing were not available (or collected) and the results of the tailored meta-analysis had not been validated.

Assessment of applicability of test accuracy studies to the review question in conventional meta-analyses relies on accurate reporting of covariates, however, additional unknown factors such as disease spectrum may cause heterogeneity which is often not measurable. This is a clear advantage of tailored meta-analysis which does not rely solely on the reported information in published studies but also draws on more specific information on the clinical setting to decide which studies are applicable. This makes the overall result more plausible. As the tailored results of plausible studies using the Mahalanobis distance method was not in the applicable region and the tailored results using the binomial method was based on only three studies, we are unable to claim that the tailored result is more accurate for the primary care setting. As a result, we were unable to validate the tailored approach to meta-analysis in its current form. The tailored meta-analysis approach may require further research and development.

## Data Availability

The dataset (THIN) analysed during the current study to derive the test positive rate and the disease prevalence is not publicly available due to the data sharing agreement with the University of Birmingham on behalf of IQVIA. All other data generated or analysed during this study for the meta-analyses are included in this published article.

## Abbreviations

BRM: Bivariate random-effects meta-analysis
D: Mahalanobis distance
FC: Faecal calprotectin
IBD: Inflammatory bowel disease
MA: Meta-analysis
TMA: Tailored meta-analysis
THIN: The Health Improvement Network
ROC: Receiver operating characteristic
